# Book Reviews

**Published:** 1820-10

**Authors:** 


### CRITICAL ANALYSIS

OF

### RECENT PUBLICATIONS, IN THE DIFFERENT BRANCHES OF MEDICINE AND SURGERY;

### SELECT MEMOIRS, AND HISTORIES OF CASES;

### In the Literature of Foreign Nations.

Πατρίδες ἄρα
Ανδράσιν, ἃ πατραῖς ἄνδρες, ἀγαλλόμεθα.

*Recherches sur le Méchanisme de la Respiration et sur la Circulation du Sang.* Par ISIDORE BOURDON, Interne des Hôpitaux Civils de Paris, Elève-Naturaliste du Gouvernment. 8vo. pp. 85. Baillière, Paris; and Treuttel and Würtz, London. 1820.

THIS is a very interesting dissertation: the subjects of it have a multitude of curious, and we would almost say admirable, relations of a physiological nature; and to elucidate them, is to explain many very important things in pathology, and furnish useful indications for the management of several diseases. It is highly gratifying to those who love science for its own sake, to see one or two curious facts made the origin of such views as are here developed by M. Bourdon: the power of effecting this is the peculiar attribute of genius, and that power is accompanied in this young student with an almost equally rare qualification,—the ability to arrange a series of facts, and to conduct a train of researches, in a methodic manner. A certain mode of education will do much in this respect: M. Bourdon is a pupil of CUVIER; and a person must be very ill organized who can attend the lectures of that great man,—glowing with genius, and manifesting a power of intellectual comprehension so vast, with such a perspicuous, yet rapid, mode of generalizing an immensity of facts, that they divide the auditor's feelings between wonder and delight,—and not be able to make a decent figure with the few original observations which are comprised in this memoir.

Before we commence our analysis of this production, we should remark, that the fundamental fact to which the whole of the author's observations concentrate is not really original with him: we mean the closure of the glottis at the same time that the abdominal muscles are contracted, during the execution of various functions of the body. He himself remarks, that SENAC had stated this connection of action to take place when a person supports a great burthen on the chest; BARTHEZ, in the act of swimming; and BICHAT, in lifting or moving heavy masses. HALLER, we may add, went further, and showed, by an experiment, that the effort evaporated as it were with the escape of air from the glottis. GALEN, also, whose writings contain so many admirable physiological observations, several of which have been brought forth in our own time as original, evidently remarked the relation in question, and he has clearly pointed out its influence on several muscular actions. (See his Treatise *de Utilitate Respirationis.*) But, we must acknowledge with M. Bourdon, that the isolated facts of this kind noticed by former authors have been but obscurely seen, and have remained fruitless up to the present day. That the glottis is capable of being closed both by the falling of the epiglottis on it, and by the coaptation of the vocal cords and the arytenoid cartilages, has been proved by MAGENDIE; and this physiologist has also shown that a certain degree of contraction of it in the latter way, takes place when an animal utters acute cries: it is the object of M. Bourdon to designate the relations of this contraction with various other phenomena besides those already mentioned. He first endeavours to account for the neglect of the fact in question, or the want of any important inferences from it, from the confusion that has been thrown around it by the erroneous notions which have hitherto been entertained respecting the action of the diaphragm during certain violent muscular exertions. It has been supposed that the diaphragm *contracts* at the same time with the abdominal muscles during such efforts. Here contradictions seem to be involved; for the result of contraction of the diaphragm is *inspiration,* and how can inspiration take place if the glottis be closed? There is, besides, an evident want of object in the contraction of the diaphragm in this case; for, the glottis being closed, the air is retained in the chest, which is thereby rendered immovable, and its firmness cannot be increased by the contraction of the diaphragm. M. Bourdon has shown, in a satisfactory manner, that the diaphragm is in a state of relaxation at the time the abdominal muscles are contracted in making great muscular exertions: but we shall follow him in the order in which he has stated his observations, and advance, as he has done, the proofs of this assertion hereafter. He says, he had already made all his observations on the phenomena of respiration, when he was yet ignorant of what the greater part of physiologists had written on this subject; and he justly adds, that "a sure way to avoid many errors, when we intend to study a point in physics which is either unknown or obscure, is to follow the example of painters, to consult nature before we have recourse to the sketches which have been made of it; for every copy must be inferior to its model: the faults remain, the beauties often disappear."

He was, he says, struck in the first instance with the prompt and easy manner in which respiration is suspended on making any vigorous muscular effort; that is to say, whenever the action of the abdominal muscles are about to be employed for effecting other results than the expulsion of air from the chest; whether in vigorous exercises of the body, such as leaping, running, wrestling, swimming, &c. or in the various expulsatory efforts, as vomiting, parturition, the evacuation of the feces, &c.

He first endeavoured to ascertain what was the agent of this suspension, and proceeded in his enquiries in the following manner. On examining, individually, the organs of respiration, he felt assured, that the diaphragm, by its contraction; the glottis, by its closure; and the veil of the palate, by its elevation; could alone be capable of retaining the air in the chest, and of suspending, in consequence, the succession of the phenomena of respiration.

*Is it the diaphragm which suspends respiration?* was his first enquiry.

If the diaphragm were to continue long in contraction, it might produce this effect, as all physiologists have admitted; for, the air being introduced into the chest only by means of the contraction of this muscle, that fluid could not escape from this cavity as long as the action which brought it in persisted. The experiments made by the author to ascertain for what period its permanent action could be maintained, show it to be short; certainly not equal to the time during which a vigorous effort can be maintained, which (as will be presently shown) requires that the lungs should be dilated with air: nor can its contraction be maintained for many instants with the degree of firmness equivalent to the steadiness with which we can make a continued effort for a much longer period. If, too, the suspension of respiration in such efforts were dependent on the diaphragm, those animals who want this muscle should not have the faculty of suspending their respiration, of making those efforts which require that such a suspension should be effected. Now birds are devoid of diaphragm; and yet they exert the faculty just mentioned in flying, and in expelling their eggs. It is then certain that some other organ than the diaphragm produces, in these animals, the suspension of respiration; and it cannot be the velum palati, because they want this part as well as the diaphragm.

*Is it the veil of the palate which, in man, suspends respiration?*

In order to settle this question, the author first asked several persons who had been deprived of the velum palati by syphilis, whether they experienced any alteration of their power of passing their feces, &c.; and they all assured him, with a nasal tone, that they did not: but, not satisfied with this reply, he made them use such exertions in his presence, and he found that they were executed with as much precision as in persons having the velum palati entire. Several experiments which he made on himself served to confirm what was thus indicated, that it is not the velum palati which is the agent in the function under consideration; and that, whilst respiration is suspended, the external air has free communication with the pharynx.

*Is it the glottis which suspends respiration?*

Reasoning from its organization, he says, we should conclude that the glottis is susceptible of being exactly closed. During a great muscular effort, the larynx is raised in a certain degree; and when we at this time, alternately, in quick succession, admit the air into the lungs and partially expel it, a sound, somewhat like a *low cough,* is distinctly heard, and which evidently comes from the situation of the glottis. After making these and some other analogous observations, he resorted to some experiments on dogs, with a view to attain further evidence. The act of vomiting is that which is most readily produced in these animals when an effort of the abdominal muscles is desired; and this is excited without any cries of the animal, which are always accompanied with some degree of contraction of the glottis, and therefore would render any observation devoid of precision, with the view in question, if they occurred. Having exposed the glottis of a dog, and administered a strong emetic potion, he observed, (shortly after the appearance of the signs of nausea, as spasmodic motions of the lips, the tongue, jaws, and pharynx,) in the most distinct manner, the glottis became exactly closed at the instant when the abdominal muscles were forcibly contracted, as vomiting took place. He was assured that the epiglottis took no part in this closure. He proved the same thing in himself: after having attained the power of passing his fore-finger down to the glottis, without producing the efforts to vomit which such an attempt produces in persons in whom those parts have not been habituated to the contact of such a foreign body. M. Bourdon says he can now pass his finger into his glottis, so as to distinguish easily the summits of the arytenoid cartilages, and the folds of the mucous membrane of the larynx. When he had practised the attempts to effect this for sixty-five days, he once, on having introduced his finger more incautiously than usual, arrived suddenly at the glottis. He immediately experienced intense nausea; at the same instant, the abdominal muscles contracted, and the glottis closed: in a word, there was a real effort to vomit, though this effect did not take place. The passing of his finger to the glottis causes now only slight nausea, which can be easily withstood; and he has profited by this disposition to ascertain the state of the glottis in the muscular efforts we are in the habit of executing, either for the more extensive movements of the body, or the expulsion of the feces; and he has become assured that the mechanism is similar to that of vomiting: in all the glottis is closed, at the same time that the abdominal muscles enter into contraction. He observed, further, that the glottis was contracted by means of the meeting of the vocal cords and the arytenoid cartilages; since he distinctly felt the latter approach each other under his finger. He was certain that the epiglottis had nothing to do with the closing of the glottis, because he could keep it pressed down on the base of the tongue, without preventing that effect from taking place.

This *consensus* or *synergie*, (as the author calls this relation of action, after Barthez; and the term is certainly, in this case, more appropriate than sym*pathy*;) between the glottis and the abdominal muscles, is observed in many familiar actions of the human body; and its importance in respect to those actions will be obvious on a little consideration.

When mucous or other irritating matters are accumulated in the bronchiæ, the abdominal muscles and the glottis both concur to produce their expulsion: the former, by their contraction, communicate to the air expelled from the lungs the strong impulse which is requisite; the latter adds to the rapidity and power of the fluid, by narrowing the canal through which it has to pass. When powerful muscular movements are required, or the expulsion of substances contained in the stomach, the bladder, or the uterus, the abdominal muscles and the glottis still combine to produce those effects. " When we wish to accelerate parturition," says the author, " we urge the woman in labour to *force downwards*; that is to say, to keep silent, or, in other terms, to close the glottis, that it may prevent the expulsion of air from the lungs, and to concentrate on the uterus the action of the abdomina. muscles." M. Bourdon might have also remarked, that women generally are *unable to cry out* during the more violent expulsatory efforts in parturition: under some peculiar circumstances, they have cried out under these conditions, and it is in this case that the diaphragm has been ruptured. We may also prove the fact implied, whenever much effort is required to evacuate the contents of the intestines. Let us observe a person who, during any great efforts to overcome some mechanical obstacle, preserves total silence and retains his breath; he develops an enormous power; whilst another person, who in the same effort cries out, sings, speaks, or laughs, produces hardly any sensible results by his exertions. In the former, the glottis is closed, and his chest forms a firm *point d'appui,* fixed by the abdominal muscles, for his arms: in the latter, the glottis is more or less open, and the power of his efforts is always in an inverse ratio to the expiration which accompanies them; because here the effort of the abdominal muscles is all expended in driving the air from the chest, which they are, besides, unable to support in an equally firm manner in its contracted state, that is, when the lungs are comparatively void of air. Intestinal herniæ and evacuation of the urine have certainly taken place during violent *laughter;* but here there is alternate closure and opening of the glottis, with powerful interrupted actions of the abdominal muscles.

In a man, then, the author adds, who has no glottis, or whose trachea has an opening in it, powerful muscular motions, various habitual efforts, and especially the expulsion of the excrements when requiring much exertion, cannot be effected, unless the preternatural opening be closed on the occasion.* Several species of animals furnish evidence in support of the views just exposed. Wherever the glottis is wanting, the expulsion of matters from the body is rendered comparatively easy by some mechanism or other: either by the liquidity of the matters to be expelled, or by the absence or weakness of the sphincter muscles, or by the presence of a cloaca, in which the liquid excrements are mingled with the solid. Thus the resistances are naturally weakened where it is not possible for any great expulsive power to be developed. In those animals which possess neither lungs nor air-cells, and consequently no glottis, all the expulsions are easily made, because all the sphincters are weak, and the matters are more or less liquid. Reptiles, which have a glottis, but an imperfect one; and birds, in whom this organ is more perfect, and whose principal sphincter is also more strongly developed, form in these respects, as well as in many others, the natural link between fish and the mammalia. Amongst the latter, there are several species, as the marsupia, and especially the kangaroo, which have only rudiments of the glottis, and this disposition is accompanied with a singular structure of their principal expulsatory organs. Their uterus, bifurcated and folded, has no neck, like that of the greater part of the mammalia. From this reason the product of conception cannot remain in the uterus during the time necessary for the

* See a case of tracheal fistula related by J. L. Petit, in the *Mémoires de l'Academie de Chirurgie.*

full growth of the fetus, and it is expelled at an earlier epoch, and received into a pouch situate under the belly, which envelops the mammæ, to which the young one, still imperfectly formed, attaches itself. Although the neck of the uterus does not exist where there is no glottis, the inverse is not true, that the glottis does not exist where there is no neck of the uterus, for the glottis has other functions to perform than that of contributing to parturition.

In order to render the facts above stated more clear, and the conclusions that may be drawn from them of a more positive and general character, the author relates the results of some experiments which he made in conformity with the indications the observations above detailed presented to him. He passed into his larynx a small canula of elastic gum: he now found it not only impossible for him to produce any voice, cry out, or cough, but he also was unable to make any expulsive efforts. He tried in vain, several times, to suspend his respiration and make those efforts; as soon as the abdominal muscles contracted, the ribs were depressed, the air was evacuated from the lungs, and the viscera were hardly at all compressed: the whole effort of the abdominal muscles, and of the extensors of the trunk, vanished in producing expiration. He then stopped the canula after having made a deep inspiration, and he could, as ordinary, suspend his respiration, compress the viscera, cause redness of his face, and, in a word, produce all the phenomena of the efforts under consideration. He again left the canula open, and again he was unable to produce them. He thinks, then, that the following conclusions may be drawn from the premises:

"1°. That it is the glottis which suspends respiration during considerable efforts, in opposing, by its closure, the escape of the air contained in the lungs.

"2. Without the glottis, the action of the abdominal muscles would be constantly employed in producing expiration: neither compression of the viscera nor flexure of the trunk could be produced.

"3. There exists a real *consensus* of action between the glottis and the abdominal muscles, and, through this medium, between the glottis and the different reservoirs, the bladder, the rectum, the stomach, the uterus.

"4. The glottis does not confine its part to the production of the voice; but, by the aid of the sympathetic connexions which unite it to the abdominal muscles, charged to concur in, if not to preside over, important functions, it exerts the greatest influence on those functions themselves.

"5. Lastly, in the different efforts there is a tendency to expiration, to the production of which the closure of the glottis is an obstacle.

"Now, to establish that there is a tendency to expiration during efforts, and that respiration can be suspended only by the aid of the closure of the glottis, is it not doubly to prove that the diaphragm is not then in action?"

Although, M. Bourdon remarks, the facts already stated may, perhaps, be considered sufficient to prove this passive state of the diaphragm, yet, as the opposite opinion has hitherto been entertained by

4

physiologists, he thinks it prudent to enter into some further considerations, in order to establish this proposition.

"The certainty (he says) we have that the abdominal muscles act during efforts, furnishes one proof of the passive state of the diaphragm in these circumstances; for, to admit as possible the combined action of these organs, is to conceive that inspiration and expiration may be simultaneous, which is evidently impossible.

"2. If the diaphragm were active during efforts, it is during inspiration, as Bichat believed, that these efforts would be produced; now this is not what takes place. There is always, during efforts, either incomplete expiration, or only a tendency to expiration, conjunctures where the diaphragm is necessarily passive.

"3. When the diaphragm contracts, it descends towards the abdomen, determining the expansion of the lungs, into which the external air is transmitted. I shall, then, have proved that this organ is passive in these efforts, if I establish that the lungs are then compressed, and that the air attempts to escape from them."

To establish this last proposition, he states the facts that herniæ of the lungs form and increase during efforts; that when there is fluid in the cavity of the pleura, and which opens externally, the fluid escapes from this cavity with greatest rapidity during coughing, and when respiration is suspended: it is under the same conditions, too, that rupture of the lungs and hemoptysis, and emphysema in the external cellular tissue, are produced; that the necks of animals, as frogs and some apes, which have here extensive membraniform productions from the larynx, are tumefied; and, lastly, respiration cannot be suspended when there is a fistula in the larynx or trachea, because, as soon as the abdominal muscles contract, the air passes out of the fistulous opening. Now this, and the other facts just stated, could not take place, if the diaphragm were then contracted.

There is a case, however, in which the diaphragm may actively participate in efforts, or when these efforts may be made during inspiration: it is when the abdominal muscles contract at the very instant when inspiration begins; but this never takes place under ordinary circumstances; it is only produced in experiments; and it must have been from such cases that Araldi of Milan drew his conclusions when he asserted that expulsions were always effected during inspiration.

Having thus advanced his propositions, and, as he believes, proved them, the author enters into a regular discussion of the phenomena which take place in efforts in general, for the purpose of showing, in a more lucid and connected point of view, the parts performed by the abdominal muscles, the glottis, and the diaphragm, in those efforts. Our limits will not let us go into these details; and we trust that our abstract is sufficiently perspicuous to enable the reader to perceive, on a little reflection, all the most important of the circumstances just alluded to.

We should remark here, that though the glottis is stated above to be the *point d'appui* for the abdominal muscles during efforts, yet that this *point d'appui* may be, and really is, commonly transferred to a more remote extremity of the aërial tube in some animals, whose glottis is but imperfectly formed for the purpose in question. It is sometimes transferred to the mouth and the nostrils. This is done by several swimming mammalia, as, for example, by the *phoca;* his cheeks are puffed out and distended as he strikes his limbs. A man who swims but indifferently, or who is unpractised in the art, does the same thing; he draws up the velum palati, which closes the posterior passage to the nostrils, closes his mouth firmly, and then his cheeks are puffed out as he strikes his limbs. Many *bats* have the power of closing the *alæ nasi* firmly, and form, when flying, the necessary *point d'appui* in the same way.

Some of the experiments made by M. Bourdon, with the view to determine precisely the importance of the closure of the glottis in several efforts, are too interesting, however, to permit us to neglect to give some account of them. He made an opening into the trachea of a dog, and fixed in it a firm canula: an emetic was then administered. The animal made twelve or fifteen efforts to vomit, but without success: it was in vain that he attempted to retain his breath; the air was always rapidly expelled through the canula as soon as inspiration had terminated. The efforts to vomit seemed to be violent, and the anxiety of the animal extreme; but at length the efforts became extremely violent, respiration was short and suddenly interrupted, the whole body was in a state of tremor, and he evacuated, at repeated times, the contents of the stomach, always during expiration. This experiment was repeated several times on other dogs, and always with the same results. The author then concludes, "that the abdominal muscles powerfully contribute to produce the sort of vomiting which is effected in a rapid manner; but that, deprived of this aid, the stomach seems capable of evacuating, by its own action, the whole of what is contained in its cavity." By some observations, he has been led to believe, that the action of the abdominal muscles, without contraction of the stomach itself, produces rapid vomiting, but never completely empties the stomach.

The author took a dog who could swim well, and introduced a very long canula into an opening made in his trachea; the end of the canula fixed here was provided with a piece of leather, so as to prevent any water passing in by the wound. He put the animal into the middle of a large pond of water: the dog went to the bottom, notwithstanding all the efforts he made to swim; and he was obliged to walk along the bottom of the pond to get to the shore. The canula was sufficiently long to reach above the water.

He got a dog who could easily leap across a ditch about four feet wide. He laid bare the trachea, without making an opening into it: the dog sprang across the ditch as lightly as before the operation, to obey his master, who called him from the opposite side. M. Bourdon then opened the trachea, and placed an open canula in the wound: he then left the animal to himself; and, in his impatience to obey the voice of his master, still on the opposite side of the ditch, he exerted his efforts to leap it, as usual; but this time he fell into the middle of it, instead of clearing it, as he had always easily done before.

The author, in a second memoir, considers the influence of the expiratory actions on the circulation of the blood, chiefly with the view of showing the cause of the redness of the face, and other signs of distension of the capillary blood-vessels during expiration. HALLER explained it by the supposition, that the vessels of the lungs are in a manner folded-up during expiration. GOODWIN, from considering that the lungs were always considerably distended with air, would not admit that any obstruction to the circulation existed in this state. BICHAT went further, and said that, even though the lungs contained no air, there would be no such obstruction in them.

The author first states, that this redness of the face, &c. happens during expiration: he then proceeds to show that a temporary obstruction to the passage of the blood through the lungs takes place during violent efforts, which obstruction he thinks depends on the pressure suffered by the pulmonary vessels on the one side by means of the retained air, and on the other by the abdominal muscles. He remarks that the jet of blood from an opening in a vein is increased in force and rapidity during coughing, vomiting, &c.: the vena cava of a dog laid bare, was distended during the cries and struggles of the animal. Cases of rupture of the vena cava have happened during violent efforts; and an instance was related a few months ago in the *Journal Complementaire*, &c. of this rupture taking place during a violent cough, caused by the presence of a foreign body in the trachea. We commonly observe the jugular and other veins distended under similar circumstances. The arterial system suffers, of course, differently from the venous in the condition under consideration. During the first instants of the efforts, more blood than ordinary flows to the left side of the heart, from its being suddenly driven from the lungs with unusual violence; and hence rupture of arteries has occurred during the first moments of violent efforts: but, after this period, the large arteries have less blood than ordinary flow through them, in proportion as it is accumulated in the venous system. It is hardly necessary to state, the capillary arteries will become distended in efforts of a certain duration; this must necessarily ensue from the obstruction in the venous system.

The last experiments related by M. Bourdon were made with the object of ascertaining whether or not he could, if he chose, voluntarily produce death, by the action of his respiratory organs. That he might not give occasion to a renewal of the story of the golden tooth, he takes care to show, by references to history, that it is probable that death has been produced in this way. He first made a long expiration; he then employed all his efforts to resist the call for inspiration. At the end of thirty seconds this call had become very urgent, and at the end of fifty seconds it was irresistible.

He tried another way: he closed the glottis after a slight inspiration, and without contracting the abdominal muscles; he then tried to prevent the escape of the inspired air, and to avoid the introduction of more into the lungs. At the end of a minute the anxiety was extreme, and shortly after this the glottis opened in spite of him, the air contained in the lungs was expelled, and new inspirations then executed.

There remained another mode of effecting the same object: he made a deep inspiration, then closed the glottis, and contracted the abdominal muscles, using considerable effort. He carefully avoided temporary relaxation of the muscles. He had some of his friends near him, who were to stop him when they judged it prudent. At the end of six seconds his face was red and tumid; after twelve seconds, he experienced slight vertigo; after fifteen seconds, the vertigo had increased, and his face was of a violet colour; he saw objects as it were through a cloud; he heard the words addressed to him only in a confused manner. His friends pinched him violently, to make him stop, but he hardly felt any pain: he was on the point of losing his consciousness when his effort ceased. He doubts whether a stronger person may not be able to produce death in this way; though he thinks that no person can properly asphyxiate themselves, that is, produce death by either of the two former methods; and he makes a question of the manner in which death would take place, in the case of its being effected by means of the last experiment, whether by apoplexy, syncope, or a sort of asphyxia. The memoir concludes with the history of these philosophical vagaries.

From our account of this work, it must be evident that the author has not given it a proper title; as it treats only of *some points* in the mechanism of the circulation and respiration. By the hints in the dedication (to CUVIER) we are given to understand that it was produced in a concurrence for a prize given by the Academy of Sciences, and that another memoir gained the prize, whilst only *honourable mention* was made of that of our author. Of this we are not certain, as it not unfrequently happens that none of the memoirs is considered to deserve more than *honourable mention*, and the question is again proposed for a new concurrence. We shall soon get information on this point; and, should a *successful* work have been produced, we shall give an account of it to our readers at the earliest opportunity.

---

*Ent[illegible]rorum Synopsis, cui accedunt Mantissa duplex, et Indices locupletissimi.* Auctore C. A. RUDOLPHI, Med. et Philosoph. Doctor, &c. 8vo. pp. 811. Berolini, 1819.

THE subject of this work is one that has engaged but little attention from the generality of English physicians; we have therefore been induced to present a view of it to our readers, which will, however, be but slight and very imperfect, because it is more adapted for the study of naturalists than of medical practitioners; but we shall afterwards take up the recent Treatise of BREMSER, which is written expressly for the instruction of pathologists; and, by adducing at the same time some extracts from the estimable lectures of Professor BRERA of Padua, we expect to present the generality of English practitioners with a considerable store of new and useful information.

Prof. Rudolphi remarks, in the Preface to this work, that there is no other order of animals of which so many new species has been discovered, from the time of Linnæus to the present day, as in that of worms which inhabit the bodies of other animals. LINNÆUS, in 1767, enumerated only eleven species; GMELIN, in 1790, made them amount to two hundred and ninety-nine; ZEDER, in 1803, to three hundred; and ninety; and RUDOLPHI himself, in his former work,* described only six hundred and three species; whilst he now notices eleven hundred, comprising some that are doubtful or uncertain. The author acknowledges how much he is indebted to the recent discoveries of OLFERS and NATURER, in the Brazils, as well as to the collection in the Imperial Museum at Vienna, to the conservator of which (Dr. Bremser) he dedicates this work. We have witnessed, personally, the assiduity of Dr. Bremser in the cultivation of this department of natural history, and the zealous and polite attention he evinces towards those who desire to study the collection which he superintends; and we cannot refrain from expressing here our congratulation on his receiving this mark of respect from Prof. Rudolphi.

The work is divided into three parts: the first contains properly the *synopsis entomologica.* The author follows here the same distribution of intestinal, or more properly visceral, worms, into *orders,* as that he adopted in his former work; namely, 1°. *Nematoidea;* 2°. *Acanthocephala;* 3°. *Trematoda;* 4°. *Cestoidea;* 5°. *Cystica;* 6°. *Generis dubii vel fictitia.* The author, on describing the worms arranged in the last order, points out the errors into which some naturalists have fallen, in their additions to it; for example, he says that the *dyacanthus polycephalus* of STIEBEL is nothing else than a vegetable fragment, and that MECKELL had, in 1766, described as worms what are really the *larvæ* of some species of flies.

It would be utterly useless to enumerate all the genera and species comprised in the orders above designated, without a detailed description of the subjects of them, which would be utterly incompatible with the limits, as well as the objects, of this Journal, as such an account would be interesting only to those who are naturalists; and we shall have occasion hereafter to designate those which are found in the human body.

The second part of the work is divided into three sections; and the first is appropriated to corrections necessary to be made in the genera and species enumerated in the *Entozoologia,* and to a description of the new genera and species. The author here objects to the opinions of some other writers, that all the *hydatids* found in animals are themselves properly living animals. He confines this appellation to the species *cysticerci* and *coenuri:* excepting in those he has found no indications of peculiar animal life in any of the cystic structures found in bodies in various states of disease.

The second section treats of the anatomy and physiology of this class of animals; and the author says that he had intended to devote himself very particularly to the cultivation of these subjects; but he was drawn from it by the new and rich collection sent from the Brazils by Olfers and Naturer, an account of which he considered to be due from him. This has led him to pass over in a more imperfect and general manner than he intended the points in their history above mentioned. He discusses the observations of some eminent naturalists respecting the existence of nerves in some *entozoa,* (animals existing within others;) and he states

* *Entozoorum sive Vermium intestinalium historia.* Amstelodamii, 1808—10.

that he has himself seen the nerves of the *strongylus gigas*, described by OTTO, displayed in a very beautiful manner in a preparation of a strongylus taken from the kidney of a dog, and placed in the collection in the Museum at Pavia by SPEDALIERI. The author concludes, that the existence of nerves in some *entozoa* cannot be placed in doubt; and he expresses a desire that they should be sought for in other worms of the same order as the strongylus, namely, the *nematoidea*. OTTO supposed that he had discovered a nerve ramifying into two portions, an abdominal and dorsal, in the *ascaris lumbricoides;* but Rudolphi does not admit the filaments observed by Otto to be nerves, because the united extremity of them is lost in the integuments of the animal, and they have no real analogy in their position, their volume, or in their structure, with those discovered in the strongylus, an animal which in all the rest of its organs presents the closest similitude with other worms. He states, that what RAMDHOR supposed to be nerves in the *distoma hepaticum* are not nerves, but vascular ramifications, as GAEDE had believed, and as he has himself proved by several preparations. He says he has dissected several times with the greatest care the *amphistoma conica* and *A. triangularis*, as well as the *monostoma tenuicollo;* so large in respect to the other species, with the view of discovering some nerves in them; but without success. He expresses his doubts respecting the cerebral ganglion and the two nervous filaments which Cuvier asserts to exist in the *pentastoma tenioidea*. He says that in one which he had an opportunity of examining, which was, however, ill preserved, he did not find the distinct cerebral ganglion, and the two filaments which Cuvier stated to be nerves did not appear to him to present the appearance of nerves, but to approach rather to that of the supposed nerves in the *ascaris*.

In the third chapter of this section, the author considers the organs of nutrition of the entozoa; and, after having remarked that in these beings this function is executed in a very simple and similar manner, differing only in degree in the diverse orders, he treats particularly of those organs in the first order, and proves here that the numerous filaments by which the alimentary tube of the nematoidea is attached throughout its whole length to the skin of the animal, are really vessels destined for nutrition, and not organs subservient to respiration, as some eminent naturalists had believed. The *echinorynchus vasculosus*, (a new species,) appertaining to the order *acanthocephala*, observed by the author at Naples, led him to the discovery of the cutaneous vessels in the echynorhynchi in general, which he had not before observed, which he designates as "spectaculum amoenissimum. Vasa scilicet sunt longitudinalia, tenuia, æqualia, transversis plurimis ramosissimis ubique anastomosantia, totamque cutem perreptantia." This discovery has thrown a great light on the mode in which nutrition is effected in these animals, namely, by means of cutaneous absorption, as he had already stated in his *Entozoologia*. BREMSER has asserted that the *botriocephali* do not absorb their nourishment by the foramina about the head, but by the mouth, which is situate in the middle; which the author says may be the case with the *botriocephalus punctatus*, but that there is reason for doubting whether it be so with the other species, since the

*botriocephalus auriculatus* wants a mouth; and here the vessels of the neck, as in the tænia, all verge to, and meet in, the foramina of the head. Olfers had pretended that each joint or ring of the tænia obtained its own nutriment by cutaneous absorption, and he appropriated to this office the marginal foramina in them: the author opposes this notion; those foramina, he says, appertain to the organs of generation: but, though he denies this mode of nutrition in the entire animal, he thinks it probable that the open extremities of the nutrient vessels, which run longitudinally, may fulfil this office, in separated joints of the tænia, for each joint individually. With respect to the cystic species, the author has only observed that, in the *cysticus delphinus*, which was however but ill preserved, there were some long and very slender and delicate filaments in the tail, which appeared to him to be vessels of the kind under consideration.

In the fourth chapter, the author treats of the organs of generation, the ova and ovaria. Nothing useful can be said on this subject in an abstract, especially when graphical illustrations of verbal descriptions are wanting. This section terminates with some considerations on the life, varieties, monstrosities, and diseases, of this class of worms. Their life, he shows, is of but short duration; and shorter, he thinks, in the south of Italy than in more northern countries, when removed from the body. He supposes that there are some entozoa which are developed in one animal, and afterwards complete their life and suffer mutations in another animal; and hence he thinks some of the varieties may arise: and he adduces examples of this emigration in the *ligula*, and other species of worms, of fishes, which pass from the intestines of these to inhabit those of aquatic birds, who receive them with their food. He acknowledges, also, that visceral worms may suffer mutations in their form without changing their habitation: he did not admit this in his former work, but he has been convinced of it by the observations of Bremser on the *tænia solium*, and the *serrata*, of the dog, which he possesses devoid of the crown of little hooks on the head; and by other observations made by the same naturalist on the *echinorhynci* of aquatic birds, which, as they increase, gradually lose the spiculæ of their proboscis, as well as the sharp points situate on the body. Several instances of monstrosity are described by the author; and the chapter concludes with some notices of the morbid alterations observed by the author in various species of the *distoma*, consisting in small, hardish, round, and distinct, excrescences on the skin, and which are described in one of the plates which accompany the work.

The third section, with which this part of the volume terminates, presents a systematic and critical catalogue of the works relating to entozoa which the author had not seen, or which were not yet published in 1808, when he constructed his *Bibliotheca Entozoologica*.

There is one point in the history of the entozoa which we are not willing to enter into the consideration of; namely, that relating to their primitive origin: but we should state, that both Rudolphi and Bremser believe that they are originally formed by the materials of the animals which they inhabit, or, according to the common, but vague, and indeed unmeaning, expression, they believe that they originate by *equivocal*

*generation:* though they acknowledge that, having originated in this way, they may nevertheless be propagated by ova; and ova, as well as what are considered to be male and female organs of generation, are found in them. They also believe that these ova may pass from the bodies of the animals in which they originate, and be carried into rivers and other waters, and thence introduced into other animals of the same or of different species, and inhabit them, and suffer various modifications of their forms. Some, too, Bremser thinks, undergo alterations of form when they pass from one organ or texture of an animal to another organ or texture; and that several parts seem to produce worms which are in a manner proper to them: and he describes in his work a *lumbricus* which was taken from the nose of a woman, and which had suffered such an alteration in consequence of the change of its habitation.

The third part of the work of Rudolphi is constituted of an appendix, and of several copious indices. The appendix comprises a description of the 124 species of entozoa, many of which are new, contained in the collection sent from Brazil by OLFERS and NATURER, and the author takes care, as he goes on, to show to what part of the synopsis the enumerated species should be referred. In his general reflections on the constituents of this collection, the author remarks that many of those entozoa which are common to Europe exist also with precisely the same forms in the Brazils, whilst others of the latter country have only a more or less close analogy with those of the former; the diversities in which probably depend on the diversities of the properties of the animals which they inhabit. Neither in those sent from the Brazils, nor in those brought from other distant countries, which the author has examined, has he ever been able to find a new *genus* of entozoa.

We shall conclude this partial sketch of the Synopsis Entozoorum with an enumeration of those which inhabit the human body, accompanied with a designation of the parts to which they are in a manner proper. It must be superfluous to speak in direct terms of praise of this work, or to recommend it to the perusal of any of our readers: the talents of the author are already generally known throughout Europe, and the value of such a description as he has here given of this department of natural history, must be evident to all those who have made it an object of their study.

The following is the catalogue above alluded to.

| | |
|---|---|
| FILARIA *medinensis.* | Habitat in *contextu cellulosa generatim, muscolis, oculis.* |
| ———— *visceralis.* | . . . *glandulis bronchorum.* |
| TRICOCEPHALUS *dispar.* | . . . *intestinis crassis generatim.* |
| SPIROPTERA. | . . . *vesica urinaria.* |
| STRONGYLUS *gigas.* | . . . *renibus.* |
| ASCARIS *lumbricoides.* | . . . *intestinis tenuibus.* |
| ———— *vermicularis.* | . . . *intestino recto, colon; genitalibus externis.* |
| DISTOMA *hepaticum.* | . . . *vesicâ felleâ.* |
| POLYSTOMA *pinguicula.* | . . . *tuberculo ovarii.* |
| ———— *venarum.* | . . . *venis.* |
| BOTRIOCEPHALUS *latus.* | . . . *intestinis tenuibus.* |
| TÆNIA *solium.* | . . . *intestinis tenuibus.* |

| | | |
|---|---|---|
| CYSTICERCUS *cellulosa.* | *Hydatide continatur.* | Habitat in *muscolis, et cerebro.* |
| ——————— *visceralis.* | | Habitat in *visceribus generatim.* |
| ECHINOCOCCUS. | . . . | *visceribus generatim.* |
| *Dubii generis.* DICERAS *rude.* | . . . | *intestinis generatim.* |

### Medical and Physical Intelligence.

